# Family physician decisions following stroke symptom onset and delay times to ambulance call

**DOI:** 10.1186/1471-2296-12-82

**Published:** 2011-08-04

**Authors:** Ian Mosley, Marcus Nicol, Geoffrey Donnan, Helen Dewey

**Affiliations:** 1 National Stroke Research Institute, Melbourne, Australia; 2 Monash University, Melbourne, Australia; 3 Department of Medicine, University of Melbourne, Melbourne, Australia; 4 Department of Neurology, Austin Health, Melbourne, Australia

## Abstract

**Background:**

For stroke patients, calling an ambulance has been shown to be associated with faster times to hospital than contacting a family physician. However little is known about the impact of decisions made by family physicians on delay times for stroke patients once they have been called.

We aimed to test the hypotheses that among ambulance transported stroke patients:

• Factors associated with first calling a family physician, could be identified.

• Time to ambulance call will be longer when a family physician is first contacted.

• Medical examination prior to the ambulance call will be associated with longer delay times.

**Methods:**

For 6 months in 2004, all ambulance-transported stroke patients who presented from a defined region in Melbourne, Australia to one of three hospitals were assessed. Ambulance and hospital records were analysed. The patient and the person who called the ambulance were interviewed to obtain their description of the stroke event.

**Results:**

198 patients were included in the study. In 32% of cases an ambulance was first called. No demographic or situational factors were associated with first calling a doctor. Patients with a history of stroke or TIA were less likely to call a doctor following symptom onset (p = 0.01). Patients with a severe stroke (Glasgow Coma Scale < 9) never called a doctor first.

When a family physician was contacted (22% of cases), the time to ambulance call was significantly longer than when an ambulance was first called (p = 0.0018) (median 143 and 44 minutes, respectively). In 36% of calls to a family physician, the doctor elected to first examine the patient. Time to ambulance call was shorter when the doctor vetted the call and advised the caller to immediately call an ambulance (45%) (median 412 and 92 minutes respectively: p = 0.06).

**Conclusion:**

Time delays to ambulance call were significantly longer for stroke patients when a family physician was first contacted. Further extensive delays were experienced by patients when the family physician elected to examine the patient.

Family physicians and their staff have an important role to play in averting potential delays for stroke patients by screening calls and providing immediate advice to "call an ambulance".

## Background

Stroke is the third most common cause of death in the Australian community and the single largest cause of disability [1,2]. However little is known by the public about stroke in general, the signs and symptoms of stroke and what to do if symptoms occur [3,4]. Furthermore knowledge about the relationship between prior stroke awareness, stroke symptom recognition and time to hospital presentation is limited [5].

The management of acute stroke has been transformed by the advent of alteplase [6]. Acute stroke care now includes time critical protocols, pathways and clinical guidelines for emergency treatment [7]. Organisation of formal care processes with rapid care pathways has been shown to be associated with faster times to treatment and increased administration of alteplase [8-10]. Unfortunately such interventions cannot assist stroke patients who experience prolonged delay times in the community prior to hospital arrival.

Public awareness messages like "FAST" and "Stroke Chain of Survival" [11,12] stress community stroke symptom recognition and immediately calling an ambulance. Calling an ambulance immediately following the onset of stroke symptoms has been shown to be associated with rapid times to hospital presentation [13,14], A clinical audit undertaken by the National Stroke Foundation (Australia) in 2009 identified that 80% of patients presented by ambulance [15]. Authors of previous stroke studies have examined factors that may influence the timing of ambulance calls. These factors include patient socio-demographics, clinical factors, stroke recognition and the identity of the person who made the call [16]. It is unclear what proportion of these cases first called an ambulance or contacted others (doctor or family members) prior to an ambulance being called. Contacting a family physician may appear to members of the general public as an effective way to seek care following the onset of symptoms. However, contacting a family physician has been shown to be associated with increased delays to hospital presentation [17,18].

Little is known about the decisions family physicians make when contacted following the onset of stroke symptoms and the impact of those decisions on delay times to ambulance call, hospital presentation and appropriate treatment.

### Aims

We aimed to test the hypotheses that among ambulance transported stroke patients:

• Demographic and situational factors associated with immediately calling an ambulance, and first calling a family physician, could be identified.

• Time to ambulance call will be longer when a family physician is first contacted.

• Medical examination prior to the ambulance call will be associated with longer delay times to ambulance call.

## Methods

### Study Description

This was a prospective observational study of patients from a geographically defined region (population 383 000) in metropolitan Melbourne who presented by ambulance to one of three public hospital emergency departments (EDs) with a final ED diagnosis of stroke or transient ischemic attack.

This study region was selected for several reasons.

First, Melbourne Metropolitan Ambulance Service records for the previous 12 months indicated that 90% of all ambulance-transported stroke patients (n = 762) from this geographic region were transported to one of three hospitals, namely Austin Hospital (60%), Northern Hospital (30%), and Royal Melbourne Hospital (RMH) (10%). Second, recruitment of patients from this area via surveillance of these three hospitals was expected to yield a sample of 250 patients over a six month period, a reasonable snapshot of current practice.

Emergency department computer records at the 3 participating hospitals were used to identify potential patients for inclusion in the study. Patients were eligible for inclusion in the study if they were 18 years of age or older, were residents within the study region, were transported to hospital by ambulance, and were diagnosed by emergency department staff as having had a stroke or transient ischemic attack. Patients with subarachnoid haemorrhage were excluded.

The person who called for ambulance assistance ("the caller") was identified for each case. An investigator undertook face to face interviews with the patient and the "caller" using a semi-structured questionnaire to obtain demographic data and their description of the stroke event. Patients and callers were asked about their responses to the onset of stroke symptoms and about factors that influenced their decision to seek ambulance assistance or first contact their family doctor. The caller provided an independent account of what went on during the stroke event from the perspective of an observer unaffected by stroke symptoms. Ambulance and hospital records were analysed.

### Data Analysis

Time elapsed following the onset of symptoms was analysed in order to identify the impact of care seeking decisions on the timelines of care.

Univariate and multivariate logistic regression analyses were undertaken to explore the associations between a range of demographic, clinical, and other factors and the outcomes of immediately seeking ambulance assistance and first calling a family doctor. Variables with a univariate *P *< 0.10 were then entered into a multivariate backward stepwise linear regression model for each outcome of interest. The least significant variable was removed and the model re-run. This process was repeated until all variables had *P *< 0.05. *P *< 0.05 was considered significant. Mann-Whitney two sample rank sum tests were used to compare timelines between groups.

### Ethics Approval

Research ethics committee approval for the study was obtained from Austin Hospital, Royal Melbourne Hospital, and The Northern Hospital. The study was also approved by the Metropolitan Ambulance Service. Informed consent was sought from the patient or next of kin as appropriate and from the caller before any data were collected and interviews conducted.

## Results

Two hundred seven patients were identified as eligible for inclusion in the study. Eight patients refused to participate and one patient could not be located. One hundred ninety-eight ambulance-transported patients were recruited into the study over a 6-month period from July 9, 2004, to January 9, 2005. This represented 56% of all stroke presentations from the region. No eligible patients were recruited at RMH. Ten potential patients presented at RMH but were excluded as they were transferred by ambulance from the Northern Hospital where they first presented.

Among the 198 patients recruited into this study (45% male; mean age 79 years) 47 patients were diagnosed in the ED as Transient Ischaemic Attack (TIA), 125 Ischaemic stroke and 26 Intracerebral haemorrhage patients. Median time to ambulance call was 70 minutes (IQ15- 288).

Demographic information for included patients is shown in Table 1.

**Table 1:** Demographic characteristics of included patients

Variable	n	%
Age (mean)	79	
Age > 75	132	67
Age < 61	18	9
Male sex	89	45
Non-manual occupations (current or previous occupation)	56	28
Working (at time of event, full time or part time)	14	7
Education leaving age (median)	15	
Education: Completed high school or above	79	40
**Presenting Hospital**		
Austin	143	72
Northern	55	28
**Stroke Sub-type**		
Ischaemic Stroke (IS)	125	63
Transient Ischaemic Attack (TIA)	47	24
Intracerebral Haemorrhage (ICH)	26	13

(n = 198)

### Responses to Stroke Symptom Onset

Three common responses were identified by the patient and those with them following the onset of stroke symptoms:

• *Immediate call for ambulance assistance*. In 32% of cases (n = 64), an ambulance was called as the first response to symptoms.

• *Contacted a family physician*. In around a quarter of patients (n = 44, 22%) a family physician was contacted by phone or the patient was examined by a family physician prior to the ambulance call.

• *Contacted others (not present) prior to the ambulance call*. In these cases (n = 73, 37%) direct face to face contact was made with friends or neighbours or, phone contact was made with an external person. In each case the person contacted was not a medical practitioner or a member of staff of a medical practice.

Other responses included patients alone at onset who were unable to respond for themselves due to the severity of the stroke symptoms. These patients were subsequently found some time later by other people who then sought emergency medical assistance.

**Figure 1:**
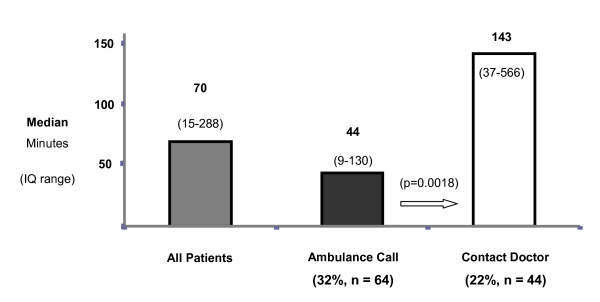
**First response and median times to ambulance call**.

Median times to ambulance call and inter-quartile ranges for each response category are included in table 2. Time to ambulance call was significantly shorter when an immediate call for ambulance assistance was made in comparison to first contacting a family physician (p = 0.0018). (Figure 1)

**Table 2:** Time in minutes from symptom onset to ambulance call by patient category (n = 198)

Response Categories	n	%	Median	Inter-quartile range	Average
All patients	198	100	70	(15-288)	405
An Immediate Ambulance call	64	32	44	(9-129)	168
Contact friends, neighbours	8	4	54	(11-105)	453
Call others	65	33	61	(21-286)	276
Contact a Family Physician	44	22	143	(27-599)	681

Factors associated with first calling an ambulance were examined. Among patient demographic variables including age, sex, living status, socioeconomic status, relationship with family physician and medical history; no factors were associated with "first calling an ambulance". Among situational variables that included stroke symptoms experienced, severity (Glasgow Coma Scale), and if the patient was alone at symptom onset; the presence of others with the patient (the patient was not alone) at symptom onset was associated with first calling an ambulance (p < 0.001). Patients with a history of stroke or TIA were less likely to first contacting a doctor (p = 0.01). Contacting a family physician was never the first response if the stroke was severe (Glasgow Coma Score of < 9).

### Response of the family physician when called

Calls to family physicians were examined to identify the impact of the doctor's response on times to ambulance call.

Doctors' responses fell into two key groups:

• The caller was advised by the doctor or a member of the doctor's staff to immediately call an ambulance.

• The patient was seen by the doctor in the doctor's rooms or the doctor visited the patient in their place of residence prior to an ambulance being called.

In 20 cases, advice was immediately given over the phone to call an ambulance by the doctor (17 cases) or by a member of the doctor's staff (3 cases). Median time to ambulance call for these patients 92 minutes (IQ 21-204). In all cases when advised to seek ambulance assistance, the caller immediately called an ambulance.

**Figure 2:**
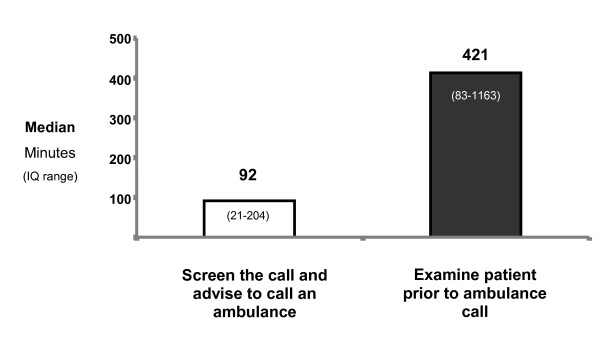
**Primary responses of doctors and median times to ambulance call**.

In 16 cases the doctor sought to first examine the patient. In nine cases the doctor visited the patient and in seven cases the patient attended the doctor's rooms. Median delay time to ambulance call was 421 minutes (IQ 83-1163) (Figure 2). There was a strong trend for longer times to ambulance call when the patient was examined first in comparison to immediate advice to call an ambulance (p = 0.06), although this was not statistically significant.

In a further eight cases the caller was unable to contact the doctor or did not wait for the doctor to arrive and called an ambulance themselves.

### Limitations

A limitation of this study is that the time of the original call to the family physician was not reported. Hence, the delay time from the initial call to the doctor to the time of the ambulance call was not available. No information was obtained about stroke patients who arrived at hospital by private transport, or from stroke patients that did not seek hospital-based care. Ten potential patients excluded from the study presented to RMH as inter-hospital transfers. This group of patients was unidentifiable in the preliminary data and may have led to an overestimation of expected patients at RMH. Data collection for this study was undertaken in 2005. Nevertheless, this study included a large representative sample of ambulance-transported stroke patients from metropolitan Melbourne and provides robust data that will inform the development of interventions to reduce delays in stroke recognition and hospital arrival.

## Discussion

This study was designed to investigate the responses to stroke symptom onset and the timelines of common responses. We found in only a third of cases the first response was to first call an ambulance. The presence of others with the patient was associated with an immediate ambulance call.

In over a third of cases calls were made to other people. The patient or those with them relied on the advice and decisions of other not present at the time prior to an ambulance being called. The authors of this study have identified previously the role of "lay referral" in the care seeking decisions of people experiencing stroke symptoms [19].

In 22% of cases the response to stroke symptoms was to contact a family physician. Time to ambulance call was significantly longer if a family physician was first contacted in comparison to first calling for an ambulance. Further, there was a strong trend for time to ambulance call to be longer again when the family physician examined the patient rather than providing immediate advice to call an ambulance. The lack of a statistically significant difference between these two groups may reflect the small numbers in this subgroup and/or common delays from symptom onset to first calling a doctor. The exact time of the doctor call was not reported.

Authors of previous studies have identified similar longer delays when family physicians were contacted [9,18]. However the impact of the doctor's response when contacted on delay times has not been previously reported.

An important finding to emerge from this study was the response of the family physician in determining the time to ambulance call and hospital arrival. Stroke patients experienced extensive delays if the doctors elected to examine them prior to calling an ambulance. Delay times were shorter when the doctor provided immediate advice to call an ambulance. Family doctors and their staff have an important role to play in averting potential delays for stroke patients by screening calls and providing advice to "call an ambulance".

In the future family physicians could be encouraged to screen calls and advise patients who may have stroke symptoms to immediately call an ambulance. Staff who take patient calls may implement a rapid assessment protocols to identify patients experiencing stroke symptoms and connect them to the doctor for immediate advice. Alternatively, the staff themselves may provide advice to call an ambulance immediately. Stroke screening tools may prompt stroke symptom recognition during patient calls and the implementation of local rapid care protocols.

Family physicians could be encouraged to screen calls and advise patients who may have stroke symptoms to immediately "Call an Ambulance".

## Conclusion

If pre-hospital delays continue to occur for stroke patients then the benefits of quality acute stroke treatments will be lost. The overall message from these findings is that the best response to the onset of stroke symptoms is to: "Call an ambulance immediately" [11].

Equally, this advice holds true for family physicians contacted following the onset of stroke symptoms. Further research is required to investigate delay times prior to hospital presentation for acute stroke patients.

## Competing interests

The authors declare that they have no competing interests.

## Authors' contributions

IM contributed to the design of the study, collected and analysed the data and led the writing of the paper. MN contributed to the design of the study, the data analysis, and contributed to the writing of the paper. GD contributed to the design of the study and the writing of the paper. HD contributed to the design of the study, the data analysis, and contributed to the writing of the paper. All authors read and approved the final manuscript.

## Pre-publication history

The pre-publication history for this paper can be accessed here:

http://www.biomedcentral.com/1471-2296/12/82/prepub
